# A Case of Unicornuate Uterus with Atypical Located Hyperstimulated Ovary after in Vitro Fertilization Pre-Embryo Transfer (IVF-ET)

**DOI:** 10.3889/oamjms.2015.069

**Published:** 2015-06-17

**Authors:** Mariya Angelova Angelova, Emil Georgiev Kovachev, Stefan Vasilev Kisyov, Vilislava Robert Ivanova

**Affiliations:** 1 *Department of Obstetrics and Gynaecology, Medical Faculty, Trakia University of Stara Zagora, Stara Zagora, Bulgaria*; 2 *Department of Obstetrics and Gynaecology, Medical University of Varna, Varna, Bulgaria*

**Keywords:** uterus unicornis, IVF, atypical located ovary

## Abstract

The authors describe a case of a congenital Mullerian anomaly, uterus unicornis with missing right fallopian tube. An in Vitro Fertilization Pre-Embryo Transfer (IVF-ET) procedure was done and presently is known that the patient has left fallopian tube and left ovary, two kidneys, and right ovary is missing. No diagnostic laparoscopy and hysteroscopy were done, only hysterosalpingography (HSG) before the IVF procedure. Several days after the follicular puncture of the left ovary the patient was urgently admitted to the hospital for specialized gynaecology in Varna. Transabdominal ultrasonography showed right ovary atypically located immediately next to the liver and with emerging theca-lutein cysts.

## Introduction

Unicornuate uterus or uterine hypoplasia occurs in agenesis or hypoplasia of one Mullerian duct. It is situated laterally and is smaller compared to the normal uterus, and loses its typical pear-like shape and obtains cylindrical shape. A typical feature is that the transverse and anterior-posterior diametre are smaller. Normally, there is one ovary at the side of location of the uterus. Two main varieties can be found, a rudimentary horn with communication or without communication, and also without a rudimentary horn. The latter variant can be found in aplasia of one Mullerian duct and accounts for 7-13 % of the Mullerian anomalies [1-3]. Uterus unicornis involves serious reproductive problems which are often associated with urological anomalies (renal agenesis, pelvic kidney). The presence of uterine horn without communication can be a major clinical problem, with formation of haematometra and subsequent surgical excision. According to literature, incidence of miscarriage is between 40% and 45%, and premature births are about 20% [2, 4]. In this anomaly incidence of primary sterility is between 5 – 20%.

The aim of this study was to describe a case of congenital Mullerian anomaly, uterus unicornis with missing right fallopian tube.

## Clinical case

**Figure 1:**
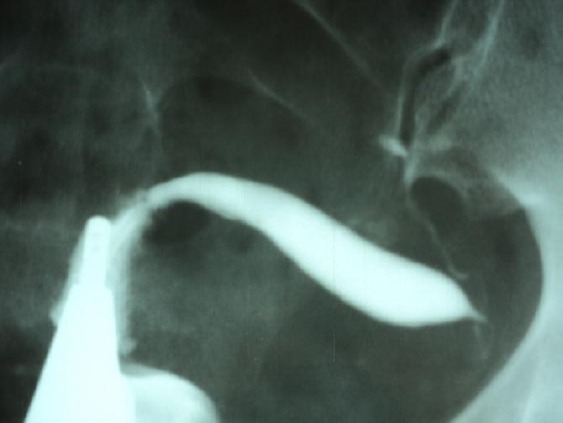
*Unicorn uterus with left tube missed scare contrast*.

This is a 36-year-old patient with primary sterility, with HSG indications of uterine anomaly (uterus unicornis), with left tube (Fig. 1) and left ovary imaged by ultrasonography, normal spermiogram. Right tube was missing and no right ovary was imaged by transvaginal ultrasonography. An IVF procedure was done at a medical centre in the capital city of Sofia on 9.11.2013 by follicular puncture and aspiration of the left ovary. Two egg cells were identified and one embryo developed and was frozen due to pronounced abdominal discomfort of the patient who in the meantime left Sofia. The patient was urgently admitted on the night of 13.11.2013 in Prof. Dr. D. Stamatov Hospital for Specialized Obstetrics and Gynaecology in Varna, Case No 6433, with severe colic like pains in the hipogastrium and collapsic symptoms.

**Figure 2:**
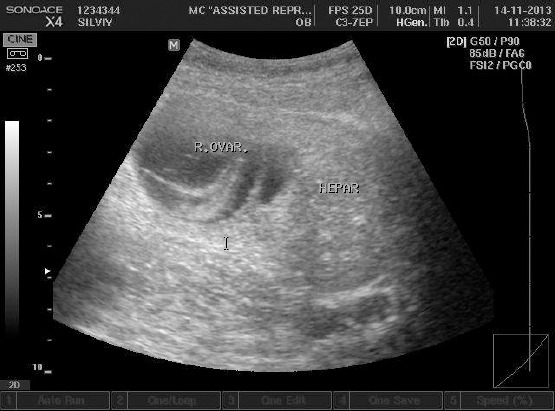
*Right ovary and liver*.

**Figure 3:**
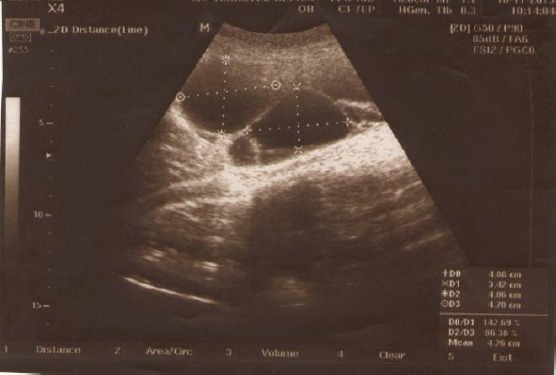
*Teca lutein cysts of the right ovary*.

Ovarian torsion after IVF was suspective, as well as development of ovarian hyperstimulation syndrome. Transabdominal ultrasonography showed presence of scarce quantity of ascites, but much to the surprise of the authors of this study and patient herself, also a right ovary was imaged, atypical located immediately next to liver (Fig. 2), and presence of two kidneys. Emerging theca-lutein cysts and ovarian enlargement were noticed (Fig. 3).

The right ovary was not possible to be imaged by transvaginal ultrasonography and therefore the colleagues who performed the IVF procedure failed to notice it.

**Figure 4:**
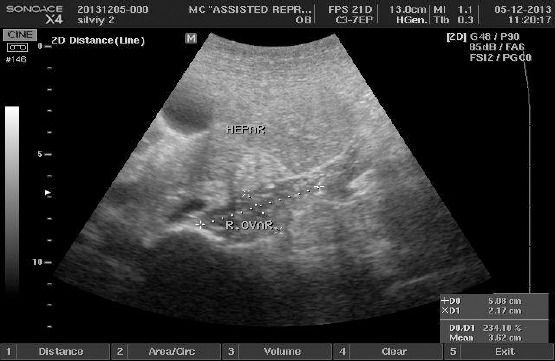
*Right ovary normal size*.

The patient was under dynamic monitoring, continuous screening of clinical and laboratory parameters, and also infusion and anticoagulant treatment was administered while placed in the Gynaecological Ward. Due to the intimate proximity of the right ovary to the liver and the risk of rupture of the theca-lutein cysts with subsequent intra-abdominal bleeding, an abdominal surgeon was consulted for eventual surgical intervention. Daily ultrasound scans were used to assess size of ovary, cystic formations and quantity of ascites. The patient was discharged on the tenth day of hospitalization in good general condition and minimal abdominal discomfort. Examination on 05.12.2013 showed normal right ovary without presence of cystic formations and no ascitic fluid (Fig. 4).

## Discussion

Congenital anomalies of the Mullerian ducts are extremely diverse and sometimes existing classifications find it difficult to categorize the specific anomaly or make it incorrectly. In the present case there are serious gaps in the diagnosis made before the IVF procedure, no diagnostic laparoscopy and hysteroscopy were done to obtain greater detail of the type of anomaly, and to image the atypical located ovary. In this case it is not clear whether a future pregnancy is possible and successful, and also the relevance of the implemented IVF procedure. HSG provides fast imaging of the shape, size and position of the uterus, and most particularly the condition of the fallopian tubes, however, an uncertain diagnosis requires the application of endoscopic diagnostic and treatment methods to differentiate the type of Mullerian anomaly and also to establish hydrosalpinx and suspective peritubular adhesions [6].

In combined genitourinary anomalies magnetic resonance is an option. Three-dimensional ultrasonography is a quick method that gives good images of the uterus and its cavity in coronal plane, best demonstrating its shape, and tubular ostiums and cervical canal. The method is very effective and useful in the diagnosis of congenital anomalies of the uterus [5]. In some uterine anomalies, the only real alternative is the carrying of surrogate pregnancy which unfortunately is not yet regulated by law in Bulgaria.
